# Olfactory Stimulation and the Diagnosis of Patients With Disorders of Consciousness: A Double-Blind, Randomized Clinical Trial

**DOI:** 10.3389/fnins.2022.712891

**Published:** 2022-02-17

**Authors:** Jing Wang, Shaoming Zhang, Wenbin Liu, Yao Zhang, Zhouyao Hu, Ziwei Sun, Haibo Di

**Affiliations:** ^1^International Unresponsive Wakefulness Syndrome and Consciousness Science Institute, Hangzhou Normal University, Hangzhou, China; ^2^Shanghai Yongci Rehabilitation Hospital, Shanghai, China

**Keywords:** disorders of consciousness, minimally conscious state, olfactory stimulation, diagnosis, prognosis

## Abstract

**Objectives:**

The aim of this study was to determine whether behavioral responses elicited by olfactory stimulation are a predictor of conscious behavioral response and prognosis of patients with disorders of consciousness (DOC).

**Methods:**

Twenty-three DOC patients (8 unresponsive wakefulness syndrome [UWS]; 15 minimally conscious state [MCS]) were recruited for this study in which 1-Octen-3-ol (familiar neutral odor) and pyridine were used to test odor behavioral responses, and water was used as an odorless stimulus. One rater presented the three odors in front of each patient’s nose randomly, and another one videotaped all behavioral responses (e.g., pouting, wrinkling nose, slightly shaking head, frowning, etc.). Two independent raters, blind to the stimuli and the patient’s diagnosis, gave the behavioral results according to the recorded videos. One-, 3-, and 6-month follow-up evaluations were conducted to obtain a good prognostic value.

**Results:**

All MCS patients showed behavioral responses to the 1-Octen-3-ol stimulus; nine MCS and one UWS showed olfactory emotional responses to the pyridine, and two MCS showed olfactory emotional responses to the water stimulus. The incidence of behavioral response was significantly higher using 1-Octen-3-ol than it was for water by McNemar test (*p* < 0.001), significantly higher using pyridine than it was for water (*p* < 0.01). The χ^2^ test results indicated that there were significant differences between MCS and UWS to 1-Octen-3-ol (*p* < 0.001). For MCS patients, the incidence of behavioral response was no different between using 1-Octen-3-ol and pyridine (*p* > 0.05). There was no significant relationship between the olfactory behavioral response and the improvement of consciousness based on the χ^2^ test analysis (*p* > 0.05).

**Conclusion:**

Olfactory stimuli, especially for the familiar neutral odor, might be effective for eliciting a conscious behavioral response and estimating the clinical diagnosis of DOC patients.

**Clinical Trial Registration:**

[https://clinicaltrials.gov/ct2/show/NCT03732092], [identifier NCT03732092].

## Introduction

After a severe brain injury, there are four different stages of disorders of consciousness (DOC) (Bruno et al., 2011). The patient usually remains in a coma for several days to several weeks. When a patient awakens from a coma (i.e., open the eyes) yet remains responsive (i.e., only show reflex movement), which is known as unresponsive wakefulness syndrome (UWS) (Laureys et al., 2010). While there is a complete lack of awareness by these patients regarding themselves or the environment, they exhibit no directional response to external stimuli. However, when a patient has a repeatable non-reflective response, it is suggested that they have entered a minimally conscious state (MCS) (Giacino et al., 2002). MCS is also subcategorized based on the complexity of a patient’s behavior (Bruno et al., 2011) as follows: MCS minus (MCS^–^) describes lower-level behavioral responses (i.e., visual pursuit and localization of noxious stimulation and contingent behavior, such as appropriate smiling or crying, to emotional stimuli), and MCS plus (MCS^+^) describes higher-level behavioral responses (i.e., command following, intelligible verbalizations, or nonfunctional communication). Once the patient shows that they can either perform functional communication or functionally use items, the diagnosis becomes emergence from a minimally conscious state (EMCS). It is difficult to evaluate bedside consciousness in DOC patients clinically, although the development and application of the Coma Recovery Scale–Revised (CRS-R) have significantly reduced the misdiagnosis rate of conscious (Kalmar and Giacino, 2005; van Erp et al., 2015; Zhang et al., 2019; Wang et al., 2020). In CRS-R, six subscales are used to evaluate patients from multiple sensory channels, including auditory, visual, motor, oromotor, verbal, communication, and arousal, to reduce misdiagnoses due to the perceived deficits of patients (Giacino et al., 2004; Kalmar and Giacino, 2005). Patients with coma, UWS, MCS^–^/MCS^+^, and EMCS can be distinguished by CRS-R.

Olfaction can be directly projected to the cerebral cortex without being transferred by the thalamus, and olfactory receptors are implicated in saliency processing and memory (involving the amygdala, hippocampus, etc.) (Smith, 2008). The orbitofrontal cortex is activated significantly when individuals have recalled smells of great personal significance. It is also involved in the formation and extraction of autobiographical memories unrelated to olfactory stimuli (Chu and Downes, 2002; Watanabe et al., 2018). Compared with visual and auditory cues, olfactory cues are more effective in inducing autobiographical memories and affecting autonomic nervous system activities, cognition, and behavior (De Bruijn and Bender, 2018).

The most expressive way that humans display emotions is through facial expressions. Per a person’s emotional experience, most psychologists divide emotions into six primary categories: happiness, sadness, fear, anger, surprise, and disgust (Cohen et al., 2003). Other emotions are composed of these six basic emotions. Studies have found that pleasant smells can induce positive emotional states, whereas bad smells can lead to negative emotional states (Vernet-Maury, 1999) and even affect cognition (Pause et al., 2004; Chen et al., 2006; Prehn et al., 2006; Zhou and Chen, 2009). Newborns show significant facial and respiratory changes, even at low concentrations of olfactory stimuli; they can distinguish between good and bad smells and exhibit aversion to bad smells (wrinkling nose, pouting) (Soussignan, 1997; Bensafi et al., 2002). These explicit manifestations have become indicators for observing and judging emotional changes in these previous olfactory studies.

In a recent functional magnetic resonance imaging study, most UWS patients and all MCS patients showed significant preservation of olfactory neural processing (Nigri et al., 2016). In addition, most MCS patients showed significant activation in higher-order olfactory processing associated with the conscious experience of odor stimuli. However, there are no studies on behavioral responses (such as pouting, wrinkling nose, shaking head slightly, frowning, etc.) elicited by olfactory stimuli in DOC patients. From the results of these previous studies, the use of olfactory stimuli seems vital for DOC patients’ clinical behavioral assessment and prognosis.

Thus, the aim of this study was to determine whether behavioral responses elicited by olfactory stimulation are a predictor of consciousness and good conscious recovery. The first hypothesis was that olfactory stimulation might effectively produce a conscious behavioral response in clinical bedside assessments. The second hypothesis was that DOC patients’ olfactory behavioral responses (e.g., wrinkling nose, pouting, etc.) might indicate the patient’s prognosis (conscious improvement).

## Materials and Methods

### Participants

Patients were recruited from the neurology unit of the Shanghai Yongci Rehabilitation Hospital (Shanghai, China) and the rehabilitation unit of Wujing Hospital of Hangzhou (Zhejiang, China).

The inclusion criteria were as follows: (1) age ≥ 18 years old; (2) no administration of neuromuscular blockers or sedation within 24 h of enrollment; (3) a diagnosis of UWS or MCS, based on repeated behavioral assessments using the CRS-R (i.e., at least five assessments within one week) (Wannez et al., 2017); and (4) no tracheotomy. The exclusion criteria were as follows: (1) coma; (2) psychiatric or neurological illness; (3) neuromuscular blocking agents or sedative drugs administered within the prior 24 h; (4) a documented history of a prior coma, critical illness, or unstable medical condition; and (5) an open tracheotomy state.

In total, 23 DOC patients were recruited for this study (7 females/16 males; aged 22 to 69 years; time since injury: 1–11 months). Ten patients had suffered a traumatic brain injury (TBI) (e.g., DOC was caused by a car accident, a fall from a high place, etc.), and 13 had suffered a non-TBI (NTBI) (e.g., DOC was caused by stroke, anoxia, etc.). Of the 23 DOC patients, eight were diagnosed with UWS (three females/five males; aged 34-69 years; time since injury: 4–10 months; three TBI/five NTBI), and 15 were diagnosed as MCS (4 females/11 males; aged 22–66 years; time since injury: 1–11 months; six TBI/nine NTBI). Demographic and clinical data of the 23 DOC patients are shown in the Supplementary Table.

The present study was approved by the Ethical Committee of Hangzhou Normal University. The patients’ relatives and caregivers were informed about the experimental procedure, after which they signed written informed consent. This study was conducted according to the World Medical Association’s Declaration of Helsinki.

### Study Procedure

Two kinds of odorant, 1-Octen-3-ol (the odor quality of a mushroom, a neutral odorant, which has been used in the previous study) (Nigri et al., 2016) and pyridine (distinctive fish-like smell, an unpleasant odorant), were used as the sensitive odors in this study. Besides, water was used as an odorless stimulus (odorless condition). We used water as the third stimulus to exclude behavioral responses elicited by visual stimulus. Using the same type of container to hold these three liquids, the packaging of the three containers was consistent and marked with A, B, and C labels, respectively. The raters did not know which odor the labels represented.

All patients were assessed in a sitting position and were free of sedative drugs. Experienced raters evaluated the level of consciousness by using the standard CRS-R at least five assessments within 1 week. Besides, the olfactory stimuli behaviors were assessed by two other raters (well-trained and experienced in the CRS-R and DOC). Specifically, one rater randomly presented the three odors in front of each patient’s nose and 5 s for each odor. The odor was changed after 15 s, and each odor was given once. Another rater videotaped behavioral responses (e.g., pouting, wrinkling nose, slightly shaking head, frowning, etc.) to the olfactory stimulus within 10 s. Two independent raters gave the behavioral results according to the recorded videos. They were blind to the stimuli being presented and the patient’s diagnosis.

To obtain a good predictive value (i.e., conscious improvement) of patients, 1-, 3-, and 6-month follow-up evaluations were conducted *via* a CRS-R analysis after completion of the protocol. Based on the diagnosis of CRS-R, the patients emerged from MCS, MCS^–^ turned into MCS^+^, or UWS turned into MCS, and then the patient is diagnosed with conscious improvement.

Eleven items in CRS-R subscales are primarily used to identify MCS^––^/MCS^+^: consistent movement to command and reproducible movement to command in Auditory Function Scale; object recognition, object localization (reaching), visual pursuit, and visual fixation in Visual Function Scale; automatic motor response, object manipulation, and localization to noxious stimulation in Motor Function Scale; intelligible verbalization in Oromotor/Verbal Function Scale; and intentional communication in Communication Scale. Two items are primarily used to identify EMCS: functional object use in Motor Function Scale and accurate functional communication in Communication Scale.

### Statistical Analysis

An evaluation of the descriptive statistics was performed for all demographic information. Means and standard deviations were calculated for continuous variables, whereas numbers and percentages were produced for categorical variables.

Differences between the behavioral responses stimulated by 1-Octen-3-ol, pyridine, and water were analyzed using the McNemar test. The differences in the behavioral responses for stimulation between UWS and MCS were analyzed using the χ^2^ or Fisher exact tests, and the results were considered significant at *p* < 0.05. The difference between the behavioral response to olfactory stimuli and the prognosis after 6 months was analyzed *via* the χ^2^ or Fisher exact tests. Finally, we analyzed the frequency of improvement in consciousness between patients with and without olfactory behavioral responses during the 6-month follow-up evaluation.

## Results

**FIGURE 1:**
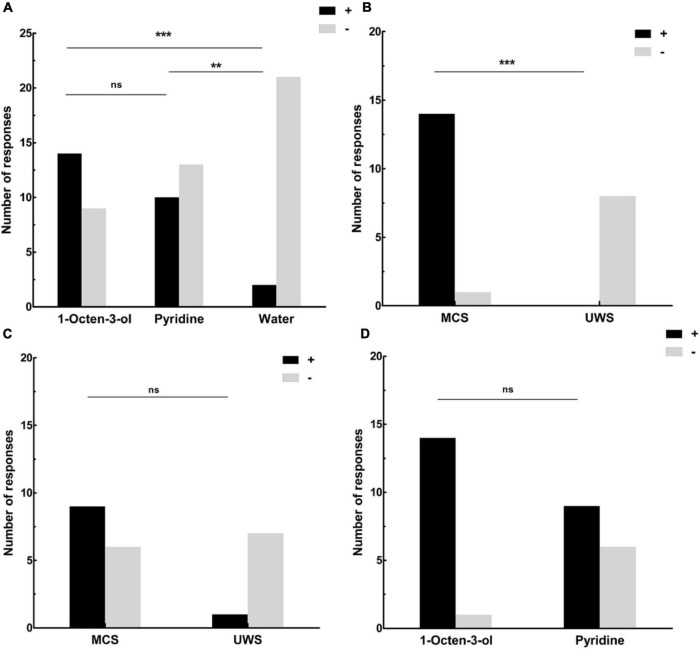
**(A)** The number of DOC patients’ responses to the olfactory and water stimuli. Fourteen showed behavioral responses to the 1-Octen-3-ol stimulus, 10 showed behavioral responses to the pyridine, and 2 showed behavioral responses to the water stimulus. **(B)** The number of responses for MCS and UWS patients to the 1-Octen-3-ol stimulus. No UWS patients showed a behavioral response to the 1-Octen-3-ol stimulus, and 14 MCS patients showed an obvious behavioral response to the 1-Octen-3-ol stimulus (93.3%). **(C)** The number of responses for MCS and UWS patients to the pyridine. One UWS patient showed behavioral response to the pyridine (12.5%), and nine MCS patients showed an obvious behavioral response to the pyridine (60%). **(D)** The number of responses to the 1-Octen-3-ol and pyridine stimuli for MCS patients. Fourteen MCS patients showed an obvious behavioral response to the 1-Octen-3-ol stimulus (93.3%). Nine MCS patients showed an obvious behavioral response to the pyridine (60%). **p < 0.01; ***p < 0.001; ns, no significant difference; black (+), behavioral responses to an olfactory stimulus; gray (–): no behavioral responses to an olfactory stimulus; UWS: unresponsive wakefulness syndrome; MCS, minimally conscious state.

Of all the DOC patients, 14 (14 MCS, 0 UWS) showed behavioral responses to the 1-Octen-3-ol stimulus, 10 (9 MCS, 1 UWS) showed behavioral responses to the pyridine, and 2 (2 MCS, 0 UWS) showed behavioral responses to the water stimulus (Table 1). The incidence of behavioral response was significantly higher using 1-Octen-3-ol than it was for water by McNemar test (χ^2^ = 10.08, degrees of freedom [*df*] = 1, *p* < 0.001), significantly higher using pyridine than it was for water (χ^2^ = 6.13, *df* = 1, *p* = 0.008), and no difference between using pyridine and 1-Octen-3-ol (χ^2^ = 1.5, *df* = 1, *p* = 0.219) (Figure 1A and Table 1).

**TABLE 1:** Different responses to different olfactory stimuli, and the information at the 6-month follow-up evaluation.

Patient	Diagnosis	Water	1-Octen-3-ol	Pyridine	6-mo Follow-up
P1	UWS	*N*	*N*	*N*	UWS
P2	UWS	*N*	*N*	*N*	MCS^–^
P3	UWS	*N*	*N*	Twisting head in avoidance	UWS
P4	UWS	*N*	*N*	*N*	UWS
P5	UWS	*N*	*N*	*N*	UWS
P6	UWS	*N*	*N*	*N*	UWS
P7	UWS	*N*	*N*	*N*	UWS
P8	UWS	*N*	*N*	*N*	MCS^–^
P9	MCS^–^	Pouting	Pouting; hitting objects with hands	Pouting; hitting objects with hands	MCS^–^
P10	MCS^–^	*N*	Pouting	*N*	MCS^–^
P11	MCS^–^	*N*	Shaking head	Shaking head	MCS^–^
P12	MCS^+^	Pushing things away with hands; grimacing	Pushing things away with hands; frowning	Pushing things away with hands; frowning	EMCS
P13	MCS^+^	*N*	Frowning; chuckling; shaking head	Pushing things away with hands	EMCS
P14	MCS^+^	*N*	Chewing	*N*	MCS^+^
P15	MCS^–^	*N*	Frowning; chewing	Frowning; chewing	EMCS
P16	MCS^+^	*N*	Twisting head in avoidance	Twisting head in avoidance; shaking head	EMCS
P17	MCS^–^	*N*	Shaking and raising the eyebrows and opening wide eyes	Raising eyebrows and opening wide eyes and staring	EMCS
P18	MCS^–^	*N*	Pouting	*N*	MCS^–^
P19	MCS^–^	*N*	Chewing, wrinkling nose	*N*	MCS^+^
P20	MCS^–^	*N*	*N*	*N*	MCS^–^
P21	MCS^–^	*N*	Putting right hand in front of nose	Swing right hand back and forth	EMCS
P22	MCS^–^	*N*	Wrinkling nose	Wrinkling nose	MCS^–^
P23	MCS^–^	*N*	Frowning; shaking head	*N*	EMCS

*EMCS, emergence from minimally conscious state; MCS, minimally conscious state; N, no response; P, patient; UWS, unresponsive wakefulness syndrome.*

When we compared the levels of consciousness with behavioral responses to the olfactory stimuli, we found that all eight UWS patients showed no behavioral response to the 1-Octen-3-ol stimulus (0%), and 14 MCS patients showed an obvious behavioral response to the 1-Octen-3-ol stimulus (93.3%). The results of the χ^2^ test indicated that there were significant differences between MCS and UWS (χ^2^ = 19.1, *df* = 1, *p* < 0.001, Fisher exact test: *p* < 0.001) (Figure 1B and Table 1). Seven UWS patients showed no behavioral response to the pyridine. One UWS patient showed an obvious behavioral response to the pyridine (12.5%), and nine MCS patients showed an obvious behavioral response to the pyridine (60%). The χ^2^ test results indicated no significant difference between MCS and UWS (χ^2^ = 4.8, *df* = 1, *p* = 0.029, Fisher exact test: *p* = 0.074) (Figure 1C and Table 1). For MCS patients, the incidence of behavioral response was no different between using 1-Octen-3-ol and pyridine (χ^2^ = 4.7, *df* = 1, *p* = 0.03, Fisher exact test: *p* = 0.08) (Figure 1D).

When we analyzed the effect of the etiology, seven TBI patients showed a behavioral response to the 1-Octen-3-ol stimulus (70%), and seven NTBI patients showed a behavioral response to the 1-Octen-3-ol stimulus (53.8%). The results of the χ^2^ test indicated that there were no significant differences among patients with different etiologies under 1-Octen-3-ol stimulus (χ^2^ = 0.619, *df* = 1, *p* = 0.43, Fisher exact test: *p* = 0.67); five TBI patients showed a behavioral response to the pyridine (50%), and five NTBI patients showed a behavioral response to the pyridine stimulus (38.5%). The results of the χ^2^ test indicated that there were no significant differences among patients with different etiologies under pyridine stimulus (χ^2^ = 0.306, *df* = 1, *p* = 0.580, Fisher exact test: *p* = 0.685).

Six months later, 10 patients (43.5%) made significant conscious improvement in behavioral diagnosis based on the CRS-R assessment (i.e., emerged from MCS, MCS^–^ turned into MCS^+^, and UWS turned into MCS at least) (Table 1). The relationship between olfactory stimuli behaviors and the prognosis of DOC patients was also analyzed. We found that 14 patients had a behavioral response to 1-Octen-3-ol; among them, 8 (57.1%) had a good outcome. Nine patients had no olfactory behavioral response; among them, two (22.2%) had a good outcome. There was no significant relationship between the olfactory behavioral response and the prognosis (χ^2^ = 2.72, *df* = 1, *p* = 0.01, Fisher exact test: *p* = 0.2). In addition, we also found that 10 patients had a behavioral response to pyridine; among them, 6 (60%) had a good outcome. Thirteen patients had no olfactory behavioral response; among them, four (30.8%) had a good outcome. There was no significant relationship between the olfactory behavioral response and the outcome (χ^2^ = 2.0, *df* = 1, *p* = 0.16, Fisher exact test: *p* = 0.22). Analyzing the predictive value of olfactory behavioral response to 1-Octen-3-ol stimulus and pyridine, the sensitivity and specificity of olfactory behavioral response to 1-Octen-3-ol stimulus were 57.1 and 77.8%, respectively. On the other hand, the sensitivity and specificity of olfactory behavioral response to pyridine were 60 and 69.2%, respectively.

## Discussion

Our research aimed to investigate the predictive utility of olfactory stimulation on consciousness and its recovery in DOC patients. This study found that no UWS patients showed a behavioral response to the 1-Octen-3-ol stimulus, but most MCS patients did show a behavioral response to the 1-Octen-3-ol stimulus. Nine MCS and one UWS showed behavioral reactions to the pyridine. The incidence of behavioral response was significantly higher using olfactory stimuli than it was for water. The incidence of behavioral response to the 1-Octen-3-ol stimulus was significantly higher in MCS patients than in UWS patients. These results support our hypothesis that olfactory stimuli are effective sensory stimulation to elicit a conscious behavioral response in clinical bedside assessments.

Facial expression recognition *via* neuroimaging and video sequences has emphasized the importance of emotion and facial expressions (Cohen et al., 2003; Herba et al., 2004). Humans show significant facial and respiratory changes, even at low concentrations of olfactory stimuli. They can distinguish between good and bad smells and exhibit an aversion to smells they dislike (wrinkling nose, pouting) (Soussignan, 1997; Bensafi et al., 2002). Moreover, fear chemosignals generate a fearful facial expression and sensory acquisition (an increased sniff magnitude and eye scanning), whereas disgust chemosignals evoke a disgusted facial expression and sensory rejection (decreased sniff magnitude, detection sensitivity, and eye scanning) (Zhou and Chen, 2009; de Groot et al., 2012). A comparison between two odors from neutral odor and unpleasant odor was used in the present study. The stimulus of 1-Octen-3-ol (neutral odor, the odor quality of mushroom) was used in previous research, which found that some UWS patients and all MCS patients showed significant preservation of olfactory neural processing by neuroimaging (Nigri et al., 2016). Furthermore, the present bedside behavioral response study found that most MCSs showed olfactory stimuli response, whereas no UWS showed it. This suggests that olfactory function is primarily preserved in patients with minimal consciousness. Recently, a study found that the olfactory response significantly distinguished between the MCS and UWS patients (Arzi et al., 2020), which also supported the present research results.

Pyridine (usually smelled in hospitals) was used as an unpleasant odor in this study. Even if most people do not like the odor, it is familiar to DOC patients, which showed a similar result with 1-Octen-3-ol in this study. Besides, the incidence of behavioral response to odorless stimulus (water) and two odorants showed that the behavioral responses might be elicited by olfactory stimulus rather than visual stimulus. Olfactory stimulation might be an effective stimulus during the behavioral evaluation in clinical diagnosis, especially for familiar neutral odors. However, one UWS patient assessed by CRS-R showed a behavioral response (twisted head in avoidance) to the pyridine, whereas there was no behavioral response to 1-Octen-3-ol. This patient was still suffering from unconscious UWS 6 months later, which might be the reflexive behavior caused by pungent odor stimulation for this patient. In this study, from the results of the standard CRS-R assessment, some of the MCS patients showed a conscious behavioral response to auditory, pain, and visual stimuli; eight patients scored on the situation behavior score table, whereas most MCS patients showed a response to neutral odor, which indicated that olfactory stimuli might be more sensitive than auditory, pain, or visual sensory stimuli during the behavioral assessment of DOC patients in the clinic. No significant prognostic value of olfactory behavioral response was concluded in the present study. Even more than half of these patients who showed olfactory behavioral response had better recovery.

Anatomically, olfaction has a specific pathway. Many scholars have deeply and extensively explored the expression of odor in the cerebral cortex and the connection between the olfactory pathway the brain’s functional areas (Zald and Pardo, 1997; Chu and Downes, 2002; Pouliot and Jones-Gotman, 2008; Rolls et al., 2010). The limbic system (including the amygdala and cingulate gyrus) is the control center of emotion and many personalized behaviors. The close relationship between odor arousal and emotion comes from the unique connection between the olfactory area of the brain’s nerve center and the amygdala and hippocampus in the limbic system, which are related to emotional arousal. This confirms that odor is the most fundamental psychological basis for emotional power (Gottfried and Zelano, 2011; Leinwand and Chalasani, 2011). Previous studies have found that the orbitofrontal cortex, amygdala, and hippocampus have some degree of functional impairment in patients with DOC (Laureys et al., 1999; Stender et al., 2014), which often leads to the depression emotions mentioned in other studies (Kotila et al., 1998; Narushima and Robinson, 2002). This may also be why the patient’s emotions in this study were manifested as aversion, such as frowning and shaking the head.

## Study Limitations

A limitation to this study is that there was no objective assessment of the patient’s brain function by neuroimaging, which can more accurately diagnose patients’ minimal consciousness and brain area function (Stender et al., 2014). It will be added to studies of the importance of consciousness-related items in diagnosing MCS. In the future, neuroimaging or/and electrophysiological methods can be used to analyze the correlation between brain function and olfactory behavioral responses. In addition, the sample included only 23 patients. Further investigation with a larger sample needs to be done to validate our findings.

## Conclusion

In conclusion, our study emphasized that olfactory stimuli, especially for the familiar neutral odor, might effectively elicit a conscious behavioral response and estimate the clinical diagnosis of DOC patients.

## Data Availability Statement

The raw data supporting the conclusions of this article will be made available by the authors, without undue reservation.

## Ethics Statement

The studies involving human participants were reviewed and approved by the Ethical Committee of Hangzhou Normal University. The patients/participants provided their written informed consent to participate in this study.

## Author Contributions

ZH, ZS, JW, WL, and SZ substantially contributed to the acquisition of data. ZH and JW substantially contributed to the analysis of data. YZ, JW, ZH, and HD substantially contributed to the interpretation of data. HD substantially contributed to study supervision. All authors contributed to the article and approved the submitted version.

## Conflict of Interest

The authors declare that the research was conducted in the absence of any commercial or financial relationships that could be construed as a potential conflict of interest.

## Publisher’s Note

All claims expressed in this article are solely those of the authors and do not necessarily represent those of their affiliated organizations, or those of the publisher, the editors and the reviewers. Any product that may be evaluated in this article, or claim that may be made by its manufacturer, is not guaranteed or endorsed by the publisher.
