# Supplementary material for: Olfactory Stimulation and the Diagnosis of Patients With Disorders of Consciousness: A Double-Blind, Randomized Clinical Trial
Source: Front Neurosci. 2022 Feb 17;16:712891. doi: 10.3389/fnins.2022.712891 (PMC8891647; doi:10.3389/fnins.2022.712891)
Supplement: Supplementary file 1 [file Table_1.DOCX]

**SUPPLEMENTARY TABLE |** Demographic and clinical data of all patients.

| Patient | Gender | Age | Etiology | Month after injury | Diagnosis | Subscale scores (A-V-M-O/V-C-Ar) | Situational behavior score table of CRS-R |
| --- | --- | --- | --- | --- | --- | --- | --- |
| P1 | Female | 44 | TBI | 4 | UWS | 102101 | - |
| P2 | Female | 34 | NTBI | 5 | UWS | 012002 | - |
| P3 | Male | 53 | NTBI | 4 | UWS | 002101 | - |
| P4 | Male | 46 | TBI | 6 | UWS | 102102 | - |
| P5 | Male | 69 | NTBI | 4 | UWS | 112102 | - |
| P6 | Male | 66 | TBI | 5 | UWS | 112102 | - |
| P7 | Female | 63 | NTBI | 10 | UWS | 212102 | - |
| P8 | Male | 55 | NTBI | 8 | UWS | 102102 | - |
| P9 | Male | 65 | NTBI | 3 | MCS- | 232102 | - |
| P10 | Female | 43 | NTBI | 4 | MCS- | 132102 | - |
| P11 | Female | 39 | TBI | 4 | MCS- | 132102 | - |
| P12 | Male | 66 | NTBI | 3 | MCS+ | 445102 | + |
| P13 | Male | 22 | TBI | 3 | MCS+ | 345102 | + |
| P14 | Female | 48 | TBI | 6 | MCS+ | 235112 | + |
| P15 | Male | 61 | NTBI | 7 | MCS- | 245102 | + |
| P16 | Female | 34 | TBI | 4 | MCS+ | 353212 | + |
| P17 | Male | 52 | NTBI | 11 | MCS- | 115102 | - |
| P18 | Male | 60 | NTBI | 1 | MCS- | 232102 | - |
| P19 | Male | 64 | TBI | 4 | MCS- | 235102 | + |
| P20 | Male | 64 | NTBI | 6 | MCS- | 132102 | - |
| P21 | Male | 54 | TBI | 3 | MCS- | 145102 | + |
| P22 | Male | 59 | TBI | 8 | MCS- | 115102 | - |
| P23 | Male | 40 | NTBI | 2 | MCS- | 135102 | + |

P, patient; TBI, traumatic brain injury; NTBI, non-traumatic brain injury, UWS: unresponsive wakefulness syndrome, MCS: minimally conscious state; +: showing the situational behavior; -: not showing the situational behavior; A-V-M-O/V-C-Ar: Auditory-Visual-Motor-Oromotor/Verbal-Communication-Arousal
